# Research Applications of Proteolytic Enzymes in Molecular Biology

**DOI:** 10.3390/biom3040923

**Published:** 2013-11-08

**Authors:** János András Mótyán, Ferenc Tóth, József Tőzsér

**Affiliations:** Department of Biochemistry and Molecular Biology, Faculty of Medicine, Medical and Health Science Center, University of Debrecen, POB 6, Debrecen H-4012, Hungary; E-Mails: motyan.janos@med.unideb.hu (J.A.M.); tothfree@gmail.com (F.T.)

**Keywords:** proteolytic enzymes, proteases, molecular biology research applications

## Abstract

Proteolytic enzymes (also termed peptidases, proteases and proteinases) are capable of hydrolyzing peptide bonds in proteins. They can be found in all living organisms, from viruses to animals and humans. Proteolytic enzymes have great medical and pharmaceutical importance due to their key role in biological processes and in the life-cycle of many pathogens. Proteases are extensively applied enzymes in several sectors of industry and biotechnology, furthermore, numerous research applications require their use, including production of Klenow fragments, peptide synthesis, digestion of unwanted proteins during nucleic acid purification, cell culturing and tissue dissociation, preparation of recombinant antibody fragments for research, diagnostics and therapy, exploration of the structure-function relationships by structural studies, removal of affinity tags from fusion proteins in recombinant protein techniques, peptide sequencing and proteolytic digestion of proteins in proteomics. The aim of this paper is to review the molecular biological aspects of proteolytic enzymes and summarize their applications in the life sciences.

## 1. Scope of the Review

Proteolytic enzymes are capable of hydrolyzing peptide bonds and are also referred to as peptidases, proteases or proteinases [1].

The physiological function of proteases is necessary for all living organisms, from viruses to humans, and proteolytic enzymes can be classified based on their origin: microbial (bacterial, fungal and viral), plant, animal and human enzymes can be distinguished.

**Figure 1:**
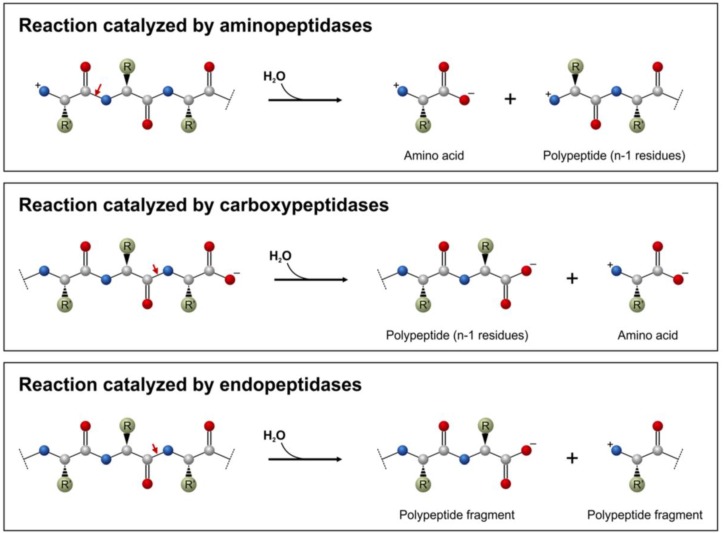
Action of aminopeptidases and carboxypeptidases removing the terminal amino acid residues as well as endopeptidases on a polypeptide substrate (having n residues). Red arrows show the peptide bonds to be cleaved.

Proteolytic enzymes belong to the hydrolase class of enzymes (EC 3) and are grouped into the subclass of the peptide hydrolases or peptidases (EC 3.4). Depending on the site of enzyme action the proteases can also be subdivided into exopeptidases or endopeptidases. Exopeptidases catalyze the hydrolysis of the peptide bonds near the *N*- or *C*-terminal ends of the substrate. Aminopeptidases (Figure 1) can liberate single amino acids (EC 3.4.11), dipeptides (dipeptidyl peptidases, EC 3.4.14) or tripeptides (tripeptidyl peptidases EC 3.4.14) from the N-terminal end of their substrates. Single amino acids can be released from dipeptide substrates by dipeptidases (EC 3.4.13) or from polypeptides by carboxypeptidases (EC 3.4.16-3.4.18) (Figure 1), while peptidyl dipeptidases (EC 3.4.15) liberate dipeptides from the C-terminal end of a polypeptide chain. Endopeptidases (Figure 1) cleave peptide bonds within and distant from the ends of a polypeptide chain [2].

Based on the catalytic mechanism and the presence of amino acid residue(s) at the active site the proteases can be grouped as aspartic proteases, cysteine proteases, glutamic proteases, metalloproteases, asparagine proteases, serine proteases, threonine proteases, and proteases with mixed or unknown catalytic mechanism [3].

The current classification system further classifies the proteases into families based on sequence similarities, furthermore, homologous families are grouped into clans using a structure-based classification [3,4]. Classification and nomenclature of proteolytic enzymes as well as a detailed description of individual proteases is available in the MEROPS database [3].

Action of the proteolytic enzymes is essential in several physiological processes, e.g., in digestion of food proteins, protein turnover, cell division, blood-clotting cascade, signal transduction, processing of polypeptide hormones, apoptosis and the life-cycle of several disease-causing organisms including the replication of retroviruses [5,6]. Due to their key role in the life-cycle of many hosts and pathogens they have great medical, pharmaceutical, and academic importance [7,8,9].

It was estimated previously that about 2% of the human genes encode proteolytic enzymes [8] and due to their necessity in many biological processes proteases have become important therapeutic targets [8]. They are intensively studied to explore their structure-function relationships, to investigate their interactions with the substrates and inhibitors, to develop therapeutic agents for antiviral therapies [9] or to improve their thermostability, efficiency and to change their specificity by protein engineering for industrial or therapeutic purposes [7]. Studying proteolytic enzymes is highly justified by their key role in several fields of industry [2,10,11,12], as well. The worldwide market of industrial enzymes was estimated to reach $3.3 billion value in 2010 and the largest segment of this market is related to proteases [13].

Proteases are extensively applied enzymes in several sectors of industry and biotechnology, furthermore, numerous research applications require the use of them, including the production of Klenow fragments, peptide synthesis, digestion of unwanted proteins during nucleic acid purification, use of proteases in cell culture experiments and in tissue dissociation, preparation of recombinant antibody fragments for research, diagnostics and therapy, exploration of the structure-function relationships by structural studies, removal of affinity tags from fusion proteins in recombinant protein techniques, peptide sequencing, and proteolytic digestion of proteins in proteomics.

This review focuses on the application of proteolytic enzymes in life sciences, especially in the field of molecular biology. The summary table of proteases discussed in this review (Table 1) contains the substrate specificities of the enzymes which are grouped based on their catalytic mechanisms.

## 2. Molecular Biology Research Applications

### 2.1. Klenow Fragment Production

The Klenow fragment is the large fragment of the *E. coli* DNA polymerase I enzyme. While the holoenzyme has 5'→3' polymerase, 3'→5' and 5'→3' exonuclease activities, the Klenow fragment has only the polymerase and the 3'→5' exonuclease activities. The Klenow fragment has several applications in the recombinant DNA technology, including the labeling, sequencing, and site-specific mutagenesis of DNA.

**Table 1:** Substrate specificity of some proteolytic enzymes used in molecular biology research. Proteases are classified based on their catalytic mechanisms, furthermore, the main sources and enzyme specificities are indicated. The arrows indicate the sites of cleavages.

Enzyme	Main source	Cleavage site
**Endopeptidases**		
*Serine proteases*		
Trypsin	bovine	-Arg or Lys↓nonspecific-
Chymotrypsin	bovine	-Trp (or Phe, Leu, Tyr)↓nonspecific-
Enterokinase	bovine	Asp-Asp-Asp-Lys↓nonspecific-
Endoproteinase Arg-C	microbial	-Arg↓nonspecific-
Endoproteinase Glu-C	microbial	-Glu (or Asp)↓nonspecific-
Endoproteinase Lys-C	microbial	-Lys↓nonspecific-
Elastase	porcine	-Ala (or Gly or Val)↓nonspecific-
Subtilisin	microbial	-Trp (or Tyr, Phe, Leu)↓nonspecific-
Proteinase K	fungal	-aromatic, aliphatic or hydrophobic ↓nonspecific-
Thrombin	bovine	-Arg (or Lys)↓nonspecific-specific for -Leu-Val-Pro-Arg-↓Gly-Ser-
Factor Xa	bovine	-Arg (or Lys)↓nonspecific-specific for -Leu-Val-Pro-Arg-↓Gly-Ser-
WNV protease	*E. coli*	-Lys (or Arg)-Arg↓Gly-Ser-
*Cysteine proteases*		
Bromelain	plant	-nonspecific↓nonspecific-
Papain	plant	-Arg (or Lys)↓nonspecific-
Ficin (ficain)	plant	-nonspecific↓nonspecific-
Rhinovirus 3C	*E. coli*	Gly-Pro dipeptide after the scissile bondhighly specific for -Leu-Glu-Val-Leu-Phe-Gln↓Gly-Pro-
TEV protease	*E. coli*	specific for -Gln-Asn-Leu-Tyr-Phe-Gln↓Gly-
TVMV protease	*E. coli*	specific for -Glu-Thr-Val-Arg-Phe-Gln↓Ser-
*Metalloproteases*		
Endoproteinase Asp-N	microbial	-nonspecific↓Asp-
Thermolysin	microbial	-Leu (or Phe)↓Leu (or Phe, Val, Met, Ala, Ile)-
Collagenase	microbial	-Pro-neutral↓Gly-Pro-
Dispase	microbial	-nonspecific↓non-polar-
*Aspartic proteases*		
Pepsin	porcine	-Phe (or Tyr, Leu, Trp)↓Trp (or Phe, Tyr, Leu)-
Cathepsin D	bovine	-Phe (or Leu)↓nonspecific (not Val, Ala)-
**Exopeptidases**		
*Serine proteases*		
Carboxypeptidase Y	yeast	-nonspecific↓nonspecific
*Cysteine proteases*		
Cathepsin C	bovine	removes N-terminal dipeptide
DAPase	porcine	removes N-terminal dipeptide
*Metalloproteases*		
Carboxypeptidase A	bovine	-nonspecific↓aromatic or branched preferred
Carboxypeptidase B	porcine	specific for C-terminal Arg or Lys

The enzymatic method to release the large protein fragment from the DNA polymerase I holoenzyme by proteolysis was published in 1970 [14]. Subtilisin-catalyzed proteolytic cleavage was used to produce Klenow fragment leading to the retention of the polymerase and the 3'→5' exonuclease activities and to the loss of 5'→3' exonuclease activity of the intact polymerase [6].

Nowadays, commercially available Klenow fragment is produced in recombinant ways in *E. coli* strains which carry the gene of large fragment of DNA polymerase I, therefore, the proteolytic production of Klenow fragment has mainly historical significance.

### 2.2. Enzymatic Peptide Synthesis

While enzymatic peptide synthesis has been frequently used to synthesize peptides for pharmaceutical and nutritional purposes (Table 2), this method is also essential for several research applications. The enzymatic method has several advantages compared to chemical methods, such as stereo specificity with side-chain protection, and the non-toxic nature of solvents coupled with the possibility of recycling the reagents used for synthesis. Enzymes have been selected considering their specificity for amino acid residues (Table 2), but this type of application is limited by the possibility of the hydrolysis of the peptide bond. The types of the enzymatic synthesis and its requirements have been reviewed [15,16,17]. Enzymatic peptide synthesis can be made by equilibrium- or kinetically-controlled methods. 

**Table 2:** Examples of peptides synthesized by proteases.

Peptide	Sequence	Enzyme(s)	Reference
Aspartame	Asp-Phe	Thermolysin	[18]
Nutritional peptide	Tyr-Trp-Val	α-Chymotrypsin, papain	[19]
Somatostatin	Ala-Gly-Cys-Lys-Phe-Phe-Trp-Lys-Thr-Phe-Thr-Ser-Cys	Thermolysin, chymotrypsin	[20]
Vasopressin	Tyr-Phe-Phe-Gln	Thermolysin, chymotrypsin	[21]
Oxytocin	Cys-Tyr Tyr-Ile Pro-Leu Leu-Gly	Papain, thermolysin, chymotrypsin	[21]
mouse EGF (21–31)	His-Ile-Glu-Ser-Leu-Asp-SerTyr-Thr-Cys	Papain, trypsin	[22]

#### 2.2.1. Kinetically Controlled Peptide Synthesis

**Figure 2:**
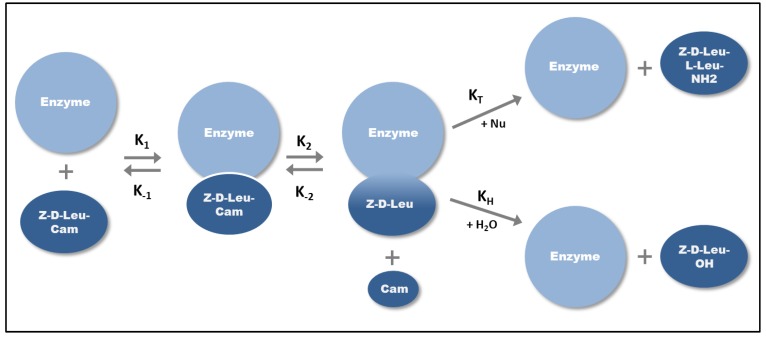
Kinetically-controlled synthesis of Z-d-Leu-l-Leu-NH_2_ dipeptide. After the formation of enzyme-substrate complex (K_1_) a covalent enzyme-substrate intermediate is formed (K_2_). The intermediate is subjected to the attack from H_2_O or other nucleophiles (Nu). K_H_ is the equilibrium constant of hydrolysis, K_T_ is the equilibrium constant of the transferase reaction.

The scheme for chymotrypsin-catalyzed kinetically-controlled Z-d-Leu-l-Leu-NH_2_ synthesis [23] is illustrated in Figure 2. The acyl donor Z-d-Leu that is activated by carbamoylmethyl (Cam) ester and chymotrypsin (E) form the enzyme-substrate complex first and after that the covalently linked Z-d-Leu-E intermediate with the loss of the carbamoylmethyl ester. If this intermediate is attacked by water, hydrolysis occurs, which results in the Z-d-Leu-OH fragment. However if a more powerful nucleophile (e.g., alcohol or thiol) is present in the media, the enzyme produces a peptide bond instead of the cleavage [15] and the Z-d-Leu-l-Leu-NH_2_ dipeptide may be formed in the presence of H-l-Leu-NH_2_ nucleophile (Figure 2). The product yield depends on the kinetics of the two nucleophilic reactions, however, the reaction is faster and requires lower substrate enzyme ratios compared to the equilibrium-controlled synthesis, due to the activated acyl donor. 

The acyl donor activating agent must not only be an ester, but an amide or a nitrile as well. Only serine or cysteine proteases can be used to perform the kinetically controlled peptide synthesis, as these enzymes can act as transferase and hence are able to catalyze the transfer of an acyl group from the acyl donor to the nucleophile through the formation of a covalent acyl enzyme intermediate. Papain, thermolysin, trypsin and α-chymotrypsin are mostly used for kinetically-controlled peptide synthesis [24]. The yield of peptide product will depend on the apparent ratio of transferase to hydrolase rate constants (K_T_/K_H_)_app_ and the rate at which the peptide product is hydrolyzed. The (K_T_/K_H_)_app_ values of proteases used for kinetically-controlled synthesis are in the range of 10^2^–10^4^ [17].

#### 2.2.2. Equilibrium-Controlled Synthesis

In the case of the equilibrium-controlled synthesis the process is the reverse of hydrolysis. Important problems of this enzymatic method of peptide synthesis are the low reaction rates and the need of increased yield because proteases do not alter the equilibrium of the reaction. A high amount of the enzyme is often required together with the precise reaction conditions to drive the equilibrium towards synthesis [15]. A higher rate of peptide bond formation can be reached by the appropriate pH of the reaction medium (changing the equilibrium of ionization) but there are several other ways to increase the yield of the reaction:

(a) Precipitation of the product is the classical method. When certain soluble carboxyl amine components are used as reactants the products will precipitate and the reduced concentration of soluble products will drive the equilibrium towards synthesis. In this case the yield of the product depends on the concentration of the starting materials and can be determined by the solubility of the product [17].

(b) In a biphasic system the enzyme acts in an aqueous environment that is surrounded by a water immiscible medium; the water content of the system is around 2–5%. The reactants (dissolved in high concentrations) can diffuse from the organic phase into the water until the equilibrium is reached; the enzyme-catalyzed synthesis is followed by the diffusion of the products back into the organic phase. The organic phase reduces the dielectric constant of the medium and thus the acidity of the carboxyl group of the acyl donor as well, which in turn promotes the synthesis of the peptide bond instead of hydrolysis [24]. This method is applicable only for the synthesis of water insoluble products.

(c) The dissolved state system can be used for the synthesis of water-soluble products (short peptides, high molecular weight peptides and proteins). In this environment forcing the reaction towards peptide synthesis requires the mass action, the addition of a water-miscible organic co-solvent in high concentration or the excess of one reactant. In serine protease-catalyzed reaction in water, the rate determining feature is the acylation of the enzyme while the product yield at equilibrium depends on the partition coefficient and the ratio of the aqueous and organic volumes [17]. The chymotrypsin- and subtilisin-catalyzed synthesis of N-Bz-L-Tyr-L-Leu-NH_2_ is more efficient in hydrophobic organic solvents; adding water in sub-saturating concentration increases the yield of the chymotrypsin-catalyzed peptide synthesis [25].

#### 2.2.3. Strategies Used in Enzymatic Synthesis

The *use of enzymes in organic solvents* have several advantages compared to aqueous solvents which have led to their widespread application: the thermodynamic equilibrium can be shifted towards synthesis, the undesirable side reactions can be reduced, the nonpolar substrates are more soluble in organic solvents, the separation process and enzyme recovery is more effective in a low water-containing environment [26,27,28,29]. Many proteases, such as thermolysin, subtilisin and α-chymotrypsin [26,29] can maintain their active conformation in organic solvents and show good functionality in the synthesis of aspartame and demorphin derivatives.

However, the use of enzymes in organic solvent has also disadvantages such as the unfavorable effects of the organic solvents on enzyme activity and stability. The *modification of biocatalysts* by protein engineering [30,31] and/or chemical modification or the use of naturally solvent-tolerant proteases [32] for peptide synthesis is a developing field. The driving force of this field is the aim of making biocatalysts with proper features to suit them for the reactions under specific synthesis conditions. Site-directed mutagenesis is a very effective tool and can be used by protein engineers to screen mutants with enhanced stability, activity or specificity, furthermore, this method can be used to explore structure-function relationships (rational design).

Subtilisin has been extensively studied and engineered via site-directed mutagenesis to make it more capable of peptide bond formation in aqueous solution [33]. Single and multiple mutations have been introduced into subtilisin to increase its stability and make it more resistant against oxidizing agents, thermal denaturations and inactivation effects of polar solvents [28].

Thermolysin has a higher synthesis rate compared to the solvent stable PST-01 protease from *Pseudomonas aeruginosa.* Considering the high structural similarity of these enzymes, the synthetic activity of PST-01 protease was increased by the Y114F mutation [31], moreover, the Y114R and Y114S mutations resulted in better activity enhancement.

Chemical modification is also an efficient method to modify the properties of enzymes used for peptide synthesis. Thiol-subtilisin, in which the serine residue has been chemically changed to cysteine at the active site, shows an enhanced aminolysis to hydrolysis ratio in aqueous solution and in dimethyl sulphoxide. The stability of proteases can also be increased by chemical modification e.g., a hydrophilic carbohydrate-polyacrylate polymer coat can make the enzymes highly active and stable in polar solvents and more resistant against thermal inactivation [28].

Immobilization of proteases is the most frequently applied method for the recovery of products without great loss of the catalysts, which greatly decreases the cost of the synthesis. This approach also ensures better operational stability of biocatalysts and control of the reaction. Enzyme immobilization techniques can be divided into five groups: (a) covalent attachment to solid support; (b) absorption on solid support; (c) entrapment in polymeric gel; (d) crosslinking with bifunctional reagents and (e) encapsulation [17,34].

*Substrate engineering* means the manipulation of the leaving group and is a powerful tool to increase the specificity of the proper enzyme and/or increase the rate and the yield of the reaction [24].

Protease-catalyzed synthesis of stereochemically modified peptides is also a preferable application compared to chemical synthesis due to stereospecificity of the proteases.

Proteases can bind not only natural substrates, but also specifically designed substrate mimetics, which are also very useful tools to increase the yield of peptide synthesis. Substrate mimetics can bind to the active site of the enzyme and in this way proteases can be used for the synthesis of products containing non-specific amino acids. The undesired cleavage of the newly synthesized peptide bonds can be avoided using this method and it is not required to change the properties of the medium or the enzyme [17].

The production of peptides with amides at their C-termini is a great challenge for enzymatic peptide synthesis, but amidation may be required to retain biological activity.

### 2.3. Nucleic Acid Isolation

Generally, the first step of nucleic acid isolation protocols is the lysis of the biological material containing the DNA or RNA of interest. Before the purification and concentration of nucleic acids the contaminating proteins and other macromolecules have to be removed from the sample. Undamaged nucleic acids can be isolated when the degradation of the DNA and RNA present in the sample is avoided by the inhibition and removal of DNases and RNases. The nucleases can be inhibited by the addition of chelators (e.g., EDTA) which bind the ions essential for their action. Besides the inactivation of nucleases, proteolytic enzymes are applied during the nucleic acid isolation to remove total protein content of the sample.

The most widely used proteolytic enzyme in nucleic acid purification is the Proteinase K, which was described in 1974 [35]. Proteinase K is a non-specific serine endopeptidase which can catalyze the cleavage of peptide bonds at the carboxylic side of aromatic, aliphatic, or hydrophobic amino acid residues. Besides the digestion of unwanted proteins, Proteinase K also quickly inactivates the nucleases which might degrade the nucleic acids present in the sample [36]. This proteolytic digestion decreases the level of contaminants in the nucleic acid extract and prevents nucleic acids from degradation leading to a higher yield of the DNA or RNA to be isolated.

### 2.4. Cell Isolation and Tissue Dissociation

Cell biology studies frequently require the dissociation of primary tissues and the isolation of viable cells for tissue culturing. The most common method for cell isolation is the enzymatic digestion of the junctions connecting the cells and the components of the surrounding extracellular matrix, by which the cells can be released from a wide variety of tissues. Several enzymes are available in the market for the detachment of cultured cells, cell dissociation and cell component or membrane-associated protein isolation [37,38]. Besides the polysaccharidases, nucleases and lipases, the proteases are the most important enzymes used widely to dissociate cells from tissues.

The experimental conditions of cell isolation are functions of several parameters, including the type of the tissue and the source of its origin. Cells with high viability can be isolated in high yield using a suitable enzyme or the optimal combination of enzymes. As proteases differ in their specificities, different enzymes are recommended to be used use for most effective tissue disruption, depending on the origin and type of the tissue. We describe below the enzymes most commonly used for cell isolation.

The matrix metalloproteinase collagenase was first isolated in 1953 [39]. This endopeptidase can digest the collagenous extracellular matrix in a zinc-dependent manner. Collagenase cleaves the peptide bonds within the triple helices of native collagen, between a neutral amino acid and Gly within the Pro-X-Gly-Pro sequence. This sequence can be found most frequently in the collagen; therefore collagenases digest other proteins less efficiently. A commercially available collagenase (clostridiopeptidase A) is produced by *Clostridium histolyticum*, and it is capable of digesting collagen fibers very effectively. Solutions supplied for tissue dissociation contain collagenase and other additional proteinases which can digest the components of the extracellular matrix [38,40]. The serine protease elastase is a unique enzyme which can cleave the peptide bonds in elastin, therefore, it is generally used to dissociate tissues containing a high amount of elastin connective fibers. Elastase cleaves peptide bonds next to smaller neutral amino acids and besides its protease activity it also has esterase and amidase activities. Papain is a cysteine peptidase of *Carica papaya* latex. Papain, similarly to elastase, also has amidase and esterase activities and has a broad specificity. Papain has less damaging effects on tissues and therefore it is typically applied for cell dissociation of neuronal tissues. Besides cell dissociation, papain is also widely used for integral membrane protein solubilization and for digestion of proteoglycans. The serine protease trypsin is a very specific proteinase cleaving the peptide bonds at the C-terminal end of positively charged Lys and Arg side chains. Due to the high specificity of trypsin the digestion of tissue proteins is less effective and it is generally used for tissue dissociation together with other proteolytic enzymes. Serine protease chymotrypsin cleaves peptide bonds preferentially at the carboxyl side of aromatic Tyr, Trp and Phe residues. Chymotrypsin is less widely used for tissue dissociation; the use of other additional proteases is required for efficient digestion. The Zn-metalloprotease dispase is also a neutral protease. This non-specific protease cleaves the peptide bonds of proteins at the amino side of non-polar amino acid residues. 

### 2.5. Cell Culturing

Cells isolated from a tissue can be cultured separately from the organism in cell culture flasks using appropriate growth medium. Adherent cells grown in a cell culture flask are attached to the surface by protein bridges which have to be disrupted during passaging. The cells can be released from the cell flask surface mechanically using a cell scraper or can be detached by a protease treatment using trypsin solution.

Trypsinization means the process used for the detachment of adherent cells using trypsin solution to digest the adhesion molecules by which the cells are attached to the surface of the culture flask. Trypsin solutions generally contain EDTA to reduce the concentration of metal ions that might inhibit trypsin.

Although trypsinization is the most commonly used method to detach adherent cells from cell culture flasks, the effects of this protease treatment were only recently studied in detail. It was found that trypsinization can affect the extracellular matrix surrounding the cells [41] and has physiological effects on cells grown in cell cultures [42]. Trypsin treatment can lead to cleavage of membrane proteins and receptors, which can cause significant changes in the expression level of different proteins: level of growth- and metabolism-related protein expressions were found to be down-regulated after trypsinization, while up-regulation of apoptosis-related protein expressions was seen after the protease treatment [42,43]. This effect should be taken into account when trypsinization is involved in experimental design.

### 2.6. Antibody Fragment Production

**Figure 3:**
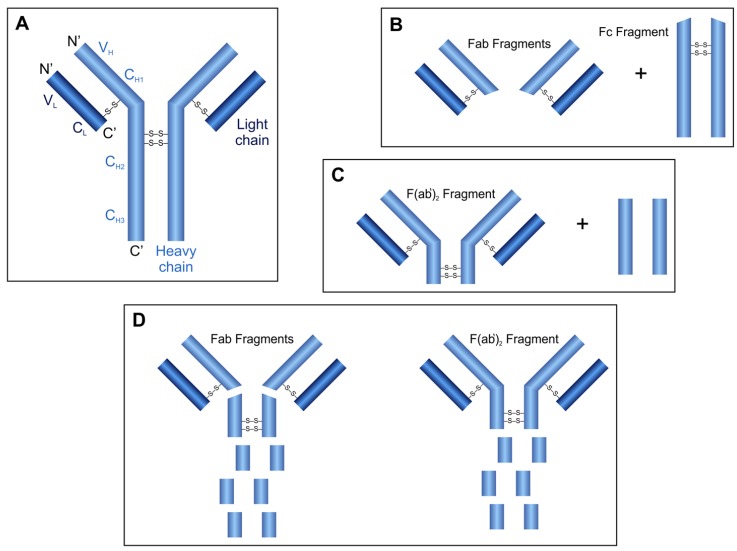
Structure of IgG antibody molecules (**A**) and fragments released after proteolytic digestion using papain (**B**), pepsin (**C**) or ficin (**D**).

Antibody molecules are produced by the immune system against foreign substances and are classified into the immunoglobulin superfamily of the proteins (Figure 3A). They consist of four polypeptide chains, two identical heavy chains (H) and two identical light chains (L) which are connected by disulfide bridges. Both the H and the L chains contain variable (V_H_ and V_L_) and constant (C_H_1, C_H_2, C_H_3 and C_L_) regions, respectively. The V_H_ and V_L_ chains, containing hypervariable regions, are responsible for the antigen-antibody interactions and determine the antigen specificity [6].

Fragments of the monoclonal antibodies are widely used in diagnostics, therapeutics and in biopharmaceutical research [44,45,46] having beneficial properties compared to the whole immunoglobulin molecules due to their smaller size and lower immunogenicity [44]. Fragments of whole immunoglobulin molecules can be produced using recombinant DNA technology or can be generated by enzymatic digestion. Here we discuss the proteolytic antibody fragmentation method.

Generally, the papain, pepsin and ficin proteases are used for the specific digestion of IgG molecules. Digestion of an antibody by the cysteine protease papain results in three fragments due to the cleavage of peptide bonds in the hinge region between C_H_1 and C_H_2 domains: one Fc (crystallizable) and two identical Fab (antigen binding) fragments are released (Figure 3B). While both released Fab fragments carry one antigen-binding site, the Fc fragment does not have antigen-binding ability. The aspartic acid protease pepsin cleaves the peptide bonds of the antibody near the disulfide bonds connecting the H chains (Figure 3C). This digestion results in the release of the peptides of the Fc region and one F(ab')_2_ fragment containing both antigen binding sites. The cysteine protease ficin can release both F(ab')_2_ or Fab fragments (Figure 3D), depending on the cysteine concentration [44].

The *in vivo* applications of the large, pentameric IgM immunoglobulines could be restricted because of their large size. Proteolytic fragmentation of IgM moleucles is also a useful method to produce smaller, active Fab, Fab', F(ab')_2_ or “IgG-like” fragments which have beneficial properties compared to the whole IgM molecules. Trypsin and pepsin are useful enzymes for IgM fragmentation, pepsin can generate Fab and F(ab')_2_ fragments, while trypsin can generate “IgG-like” fragments, as well. Papain cannot be used for IgM fragmentation because of the production of heterogeneous fragments. 

Digestion of monoclonal antibodies by papain or pepsin is still used to produce Fab or F(ab')_2_ fragments [44]. There are several products available on the market obtained by proteolytic cleavage of antibodies, furthermore, several companies supply fragmentation kits for antibody fragment preparation. In some cases it could be preferable to produce the Fab fragments in high quality by recombinant expression in cell lines and obtain the fragment in sufficient quantity. For example, the Fab fragments produced by recombinant expression were found to be more preferable for the crystallization experiments because of the higher quality of the protein sample [47].

Antibody fragments have several beneficial properties for *in vivo* applications compared to whole antibody molecules. Due to their smaller size they have higher mobility and can penetrate tissues or permeate cell membranes more easily. As antigen-binding fragments lack the Fc fragments, they have lower immunogenicity, as they contain only their antigen-binding domain sites (Fab, Fab' or F(ab')_2_ fragments) and do not carry the regions responsible for antibody effector functions, furthermore, they do not form large immunocomplexes.

The antigen-binding fragments of antibodies have great importance from the viewpoint of clinical and therapeutic applications. Antibody fragments could be administered to prevent the development of a disease (e.g., restenosis), could be applied during the diagnosis (e.g., metastatic breast and colon cancer) or for the treatment of some diseases (e.g., macular degeneration) or to detect toxins or neutralize snake venoms [44,45]. The current number of antibody-based therapeutics approved by the FDA is 35, while several other antibodies are in clinical trials. The relevance of antibody fragments in structural studies is discussed in detail in the following paragraph. Further important biotechnological applications of antibody fragments, e.g., as immunodetection, immunopurification and detoxification, have been reviewed in the recent past [46].

### 2.7. Structural Studies

Crystallographers have great challenges in the structure determination of transmembrane proteins. It is difficult to crystallize these proteins due to their high molecular flexibility, hydrophobic surfaces and low solubility. Antibody fragments are very useful tools for solving these problems. Specific binding of antibody fragments can increase the overall hydrophilicity of proteins and the solubility of the transmembrane proteins, furthermore, they can decrease the flexibility and stabilize the conformation of the molecule [48]. The structures of the membrane proteins co-crystallized with antibody fragments can be determined at higher resolution because these crystals have a higher diffraction quality [49,50,51].

The proteases have great indirect significance in structure determination, because the antibody fragments produced by proteolytic cleavage of whole immunoglobulins can be used to improve the crystallization properties [52,53,54].

The proteases are not only tools for crystallographers but are also important target molecules for structural biologists and have great relevance in antiviral therapies, drug and therapeutics development from the viewpoint of structural biology. Besides interest in proteases with unknown structure, the results of structural studies can help researchers evaluate the structure-function relationships more efficiently; increasing knowledge on the structural organization of viral proteases can help to explore their action, perform comparative studies by which we can better understand the structure-function and evolutionary relationships and recognize general or specific features [55,56,57]. One of the main driving forces of structure determination of proteases is the need for the development of efficient drugs for antiviral therapies [7,9,58,59,60]. Both structural and enzymatic inhibition studies are required for the structure-based drug development of protease inhibitors [61]. Structural data can also help protein engineers to alter the specificity and to improve the enzymatic properties of proteases by structure-guided mutagenesis [62,63,64] for several purposes.

### 2.8. Fusion Tag Removal

The proteins produced by recombinant techniques are typically linked with a fusion partner termed a fusion tag. The introduction of a fusion tag means the fusion of an additional protein or peptide to the recombinant protein. These fusion tags are extensively used from basic research to high-throughput structural biology owing to the several advantages they provide in the expression of different recombinant proteins [65,66]. The tags largely aid the detection and purification of proteins; moreover they also could have a favorable effect on protein yield and/or solubility. Tags can prevent proteins from proteolytic digestion, can protect antigenicity or facilitate the folding of the fusion protein. On the other hand, they can also negatively alter solubility, structural integrity and biological activity [47,67,68] or may cause a disadvantage for further application of the protein, so the removal of a tag can be crucial [66].

Commonly used affinity tags and fusion protein partners are the hexahistidine-tag (His_6_), FLAG-tag, maltose binding protein (MBP), glutathione S-transferase (GST), thioredoxin (TRX), small ubiquitin-like modifier (SUMO), ubiquitin (Ub) and green fluorescent protein (GFP) [69].

In some cases the tag can be removed by a chemical treatment but those methods are rather unspecific compared to enzymatic cleavages and may lead to protein denaturation and/or side chain modifications of amino acids in the target protein. The specificity and detergent sensitivity of common proteases used for tag removal have been examined and reviewed previously [70,71,72,73]. Both endo- and exo-proteases could be suitable for fusion tag removal.

#### 2.8.1. Endoproteases

Serine proteases such as enterokinase (also referred to as enteropeptidase), factor Xa and thrombin have been widely used for many years to remove N-terminal tags, but several cases have been reported in which they cleaved not only at the desired cleavage site but also in the protein of interest. These incidents led to the extensive application of viral proteases like human rhinovirus (HRV) 3C protease and tobacco etch virus (TEV) protease. While sequences recognized by a cellular serine protease and the viral proteases could be similar, the viral proteases cleave the protein substrates at the undesired sites less efficiently due to their high specificity and low catalytic rate, moreover, recombinant viral proteases can be produced in high quantities in *E. coli* [73]. These findings and the limited activity of the generally used serine proteases in some detergents, which are needed to study the membrane proteins, inspired the search for other viral proteases for tag removal, such as Tobacco Vein Mottling Virus (TVMV) protease [74], West Nile Virus (WNV) protease [75] and some alphaviral proteases: Venezuelan Equine Encephalitis Virus (VEEV) protease, Semliki Forest Virus (SFV) and Sindbis Virus (SIN) protease [76].

#### 2.8.2. Exopeptidases

Aminopeptidases and carboxypeptidases are not as widely used as endopeptidases, as they frequently leave amino acid residues on the target protein, while it is easier to design cleavage sites with endopeptidases not to leave extra residues after the cleavage. However, if is still desired to add a tag onto the C-terminal of the target protein a carboxypeptidase may be used for its removal. Among metallocarboxypeptidases, type A carboxypeptidases remove mostly aromatic or branched aliphatic side chain containing amino acids, while type B carboxypeptidases prefer basic amino acids. Carboxypeptidases can be utilized to remove a His_6_ tag from the C-terminal end of a protein [77]. Dipeptidyl aminopeptidase (DAPase) is a useful enzyme for the removal of N-terminal dipeptides.

### 2.9. Proteomic Applications

Proteomic studies are made with the aim to identify, characterize, and quantify the required samples and typically involve mass spectrometric (MS) analysis. Besides determination of the composition of protein complexes, chemical properties, post-translational modifications and structural properties of proteins can also be revealed by MS.

**Figure 4:**
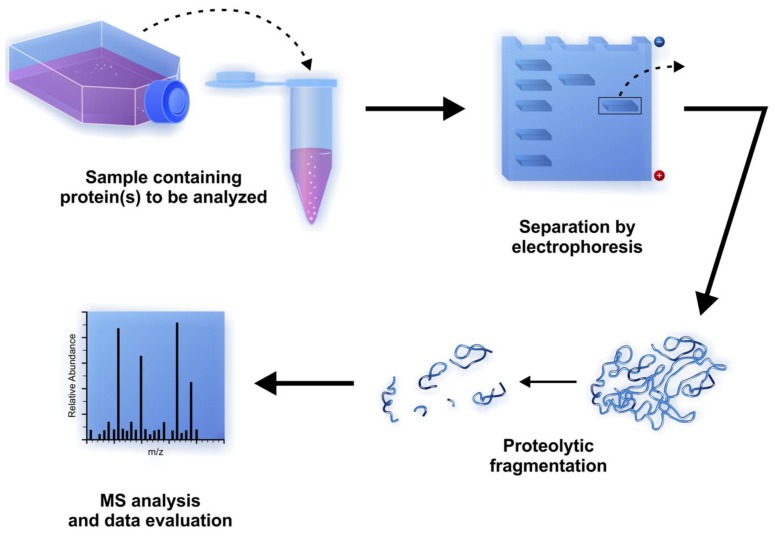
Steps of proteomic analysis using mass-spectrometry after separation and in-gel digestion of proteins of interest.

MS is a powerful analytical method to measure the mass of proteins or peptides by the analysis of mass-to-charge ratio (*m*/*z*) ratio. Generally, the samples to be analyzed contain a mixture of various proteins and/or polypeptides that have to be separated and digested into smaller fragments before the MS analysis. Protein separation can be performed efficiently by polyacrylamide gel electrophoresis. The separated proteins can be digested after the electrophoresis by chemical cleavage or by enzyme-catalyzed digestion of peptide bonds. The process in which the bands or spots are cut out from the gel followed by the addition of protease(s) to the gel containing the protein(s) of interest is called in-gel digestion [78]. Proteolysis of whole proteins leads to the release of smaller peptides with different molecular masses which are suitable for MS analysis (Figure 4).

Trypsin is the most widely used proteolytic enzyme for protein digestion in MS analysis. Due to its high specificity it is easy to predict the cleavage sites and to compare the results of experimental enzymatic and theoretical *in-silico* digestion. The Asp-N, Lys-C, Arg-C and Glu-C enyzmes are also highly sequence-specific endoproteases but they are less active [78]. Chymotrypsin, pepsin, papain, elastase, subtilisin, proteinase K, thrombin, factor Xa and some other proteases are also suitable enzymes for fragmentation in MS analysis [79].

Several proteases are available for this fragmentation of which specificities are well established [79,80]. The number and length of released peptide fragments depends on the protease(s) applied for selective proteolysis of the targeted protein. Applications developed for *in silico* protein fragmentation are useful to predict the proteolytic fragments and to choose the most proper enzyme for the most effective digestion based on the enzyme specificities (http://prospector.ucsf.edu).

Generally, highly sequence-specific proteases are preferred for protein fragmentation instead of less specific enzymes, as the latter ones produce a very complex mixture of fragments. In the case of the efficient fragmentation the peptide, the fragments have a proper length and are released in high yield, and the complete sequence of the whole protein can be covered by the analysis of the proteolytic fragments.

## 3. Summary

Besides extended application for nutritional and pharmaceutical purposes, proteases from natural sources are also widely used tools in molecular biology practice. Their degradative properties make them useful for general protein digestion in tissue dissociation, cell isolation, and cell culturing. The specificity and the predictability of cleavages by proteases enables their use for more specific tasks such as antibody fragment production, the removal of affinity tags from recombinant proteins and specific protein digestion in the proteomics field mainly for protein sequencing. Moreover, the already mentioned specificity makes proteases—in a water restricted environment—able to synthesize the peptide bonds instead of hydrolyzing them. This property combined with their enantioselectivity has also promoted their use in peptide synthesis.

The expansion of knowledge has assisted the increase of applications of proteolytic enzymes for several purposes, and the application fields are widening with the help of protein engineering techniques and by chemical modification of the enzymes [7,62]. Studies made with the aim to better understand the structure and function of existing proteolytic enzymes and to obtain new, engineered proteases with altered properties for therapeutic, industrial or research fields require the use of the applications discussed in this paper.
